# Chemotherapeutic xCT inhibitors sorafenib and erastin unraveled with the synaptic optogenetic function analysis tool

**DOI:** 10.1038/cddiscovery.2017.30

**Published:** 2017-06-19

**Authors:** Marc Dahlmanns, Eduard Yakubov, Daishi Chen, Tina Sehm, Manfred Rauh, Nicolai Savaskan, Jana Katharina Wrosch

**Affiliations:** 1 Department of Psychiatry and Psychotherapy, Friedrich-Alexander University of Erlangen-Nuremberg, Erlangen, Germany; 2 Translational Neurooncology Laboratory, Department of Neurosurgery, Friedrich-Alexander University of Erlangen-Nuremberg, Erlangen, Germany; 3 Paracelsus Medical University, Nuremberg, Germany; 4 Department of Pediatrics and Adolescent Medicine, Friedrich-Alexander University of Erlangen-Nuremberg, Erlangen, Germany; 5 BiMECON Ent., Berlin, Germany

## Abstract

In the search for new potential chemotherapeutics, the compounds’ toxicity to healthy cells is an important factor. The brain with its functional units, the neurons, is especially endangered during the radio- and chemotherapeutic treatment of brain tumors. The effect of the potential compounds not only on neuronal survival but also neuronal function needs to be taken into account. Therefore, in this study we aimed to comprehend the biological effects of chemotherapeutic xCT inhibition on healthy neuronal cells with our synaptic optogenetic function analysis tool (SOFA). We combined common approaches, such as investigation of morphological markers, neuronal function and cell metabolism. The glutamate-cystine exchanger xCT (SLC7A11, system X_c_^−^) is the main glutamate exporter in malignant brain tumors and as such a relevant drug target for treating deadly glioblastomas (WHO grades III and IV). Recently, two small molecules termed sorafenib (Nexavar) and erastin have been found to efficiently block xCT function. We investigated neuronal morphology, metabolic secretome profiles, synaptic function and cell metabolism of primary hippocampal cultures (containing neurons and glial cells) treated with sorafenib and erastin in clinically relevant concentrations. We found that sorafenib severely damaged neurons already after 24 h of treatment. Noteworthy, also at a lower concentration, where no morphological damage or metabolic disturbance was monitored, sorafenib still interfered with synaptic and metabolic homeostasis. In contrast, erastin-treated neurons displayed mostly inconspicuous morphology and metabolic rates. Key parameters of proper neuronal function, such as synaptic vesicle pool sizes, were however disrupted following erastin application. In conclusion, our data revealed that while sorafenib and erastin effectively inhibited xCT function they also interfered with essential neuronal (synaptic) function. These findings highlight the particular importance of investigating the effects of potential neurooncological and general cancer chemotherapeutics also on healthy neuronal cells and their function as revealed by the SOFA tool.

## Introduction

Malignant gliomas (glioblastomas (GBMs; WHO grades III and IV)) are primary brain tumors with lethal prognosis in adults.^1,2,3^ The median survival time from diagnosis is ~14 months.^1,3^ GBMs are hallmarked by features such as uncontrolled cellular proliferation, diffuse infiltration, and resistance to apoptosis and chemotherapy. The current standard-of-care for GBM patients includes adjuvant temozolomide treatment (brand names Temodal in Europe and Temcad in the USA).^4^ This treatment strategy is currently the best clinical practice, however, conferring still a median survival time of only 14.6 months^4^ compared with 12.2 months for patients receiving only radiotherapy.^5^

Temozolomide, or rather its metabolites, methylate DNA to inhibit tumor proliferation. However, the drug’s effects on healthy cells of the patient’s body can cause a number of adverse effects resulting from target effects as well as off-target (non-selective) effects. Temozolomide comes along with side effects, such as gastrointestinal irritations, myelosuppression, lymphophenia and opportunistic infections.^6^ This and most importantly the low 5-year survival rate demand the development of new treatment options for glioblastoma. In the hunt for new drugs, researchers aim to find compounds that are more efficient in cancer cells and more specific to them, so that they spare normal, healthy cells.

Recent evidence has spotlighted the glutamate-cystine exchanger xCT (SLC7A11, system X_c_^−^) as a potential drug target in treating glioblastoma.^7,8^ The xCT system represents a key player in glutamate, cystine and glutathione metabolism in most cells.^2,9^ xCT is highly expressed in astrocytes and has also been found in glioblastoma promoting chemotherapeutic resistance.^10^ Moreover, xCT levels are causally linked with the malignancy grade of glioblastoma.^7,11^ Apoptosis is a common form of programmed cell death that can be triggered by chemotherapeutic drugs via the intrinsic or extrinsic pathways. Recently, it has been shown that the glutamate cystine exchanger xCT appears to be essential in the process of chemo- and ferroptosis resistance in some cancer cell type.^12,13,14^ Evasion of cell death and development of redox stability are hallmarks of cancers and promote tumorigenesis as well as chemo-resistance. Since xCT plays a pivotal role in tumor microenvironment interactions, for example, in the induction of peritumoral neuronal cell death and perifocal edema,^2,11^ there is a quest for understanding the effects of inhibiting compounds for this transporter.^15^ A deeper understanding of the effects of xCT inhibition on tumor cells might lead to the development of compounds that break through these tumors’ chemo-resistance, and the elucidation of xCT-inhibitor interaction with healthy brain cells might enable us to develop compounds with less adverse, off-target effects.

The xCT inhibitor sorafenib (Nexavar, Bayer Healthcare Pharmaceuticals Inc., Whippany, NJ, USA) is currently approved by EMA and FDA for hepatocellular carcinoma,^16^ advanced renal cell carcinoma and thyroid carcinoma.^17,18^ Sorafenib is a multi-kinase inhibitor with various targets inside the cellular signaling cascades.^19^ Since a tightly regulated kinase network inside cancer cells is inevitably necessary for signal transduction and tumor growth, treatment with sorafenib impairs cell survival of these tumor cells, for example, in neuroblastoma^20^ or hepatocellular carcinoma^21^ models. With sorafenib being able to cross the blood–brain barrier it is a prospective substance to treat brain tumors, as well.^22^ In a phase II clinical trial, sorafenib was already investigated in combination with temozolomide for treatment of relapsed glioblastoma, showing high efficacy.^23^ In addition to effectively targeting the tumor cells, a promising chemotherapeutic should also target the tumor specifically. With regard to malignant brain tumors, possible unintended effects of therapeutic agents on neuronal and astrocyte functions are of particular interest.

In this study, we systematically investigate the effect of chemotherapeutically relevant concentrations of sorafenib on healthy rat hippocampal cells (mainly neurons and astrocytes). To isolate the xCT-inhibition effect of sorafenib, the experiments were conducted in parallel with erastin, a common and well-studied^8, 24,25,26,27^ model substance for xCT inhibition.

Similar to sorafenib, erastin has been recognized as xCT inhibitor with shown efficacy against tumor cell lines.^8^ Next to impairing amino acids’ transport over the membrane, erastin disrupts mitochondrial permeability transition pore (mPTP) and targets the voltage-dependent anion channel 1 (VDAC-1), mechanisms by which erastin could directly interfere with the mitochondrial energy supply of the cells.^28,29,30^ Metabolically highly active cells such as tumor cells will rapidly be depleted of required substrates and can no longer maintain cell integrity. Recently it was shown that erastin sensitizes glioblastoma cells to temozolomide, an effect that was dependent on xCT inhibition.^26,31^ Although two human studies reported psychotic disorders^32^ and an impairment of cognitive function^33^ after sorafenib treatment, the effects of sorafenib on neurons are sparsely investigated.^34,35^ Also, erastin has, to our knowledge, only been used to induce ferroptosis in immortalized hippocampal cell lines.^36^ The relevant cytotoxicity of the two substances has not been investigated up to now.

Noteworthy, many previous studies ensure neurons safety by evaluating single morphological markers.^37,38,39,40^ In some studies the amino-acid profile of treated cells has been quantified.^41,42^ In this report, we applied the synaptic optogenetic function analysis (SOFA) tool to unravel sub-morphological changes of neurons. With the SOFA tool at hand, we investigated cell survival and synaptic functional parameters in neurons treated with chemotherapeutics. We found that only with a combination of multiple viability assays and functional tools one can gain a comprehensive picture of a compound’s neurotoxic profile, especially if the effects are subtle. Key to neuronal function is the ability of neurotransmission, that is, the exocytosis of synaptic neurotransmitters, stored in synaptic vesicles, into the neural cleft where they bind to a postsynaptic receptor.^43^ The total pool of a neuron’s synaptic vesicles is well organized into different functional groups: the readily releasable pool, the recycling pool and the reserve pool.^44^ Neurons’ vesicle pool sizes are complex and highly regulated systems. Its dynamics are part of presynaptic plasticity and an unbalanced homeostasis is involved in different psychiatric and neurological disorders.^45,46,47,48^ Major depressive disorder—one of the common adverse effects of glioblastoma treatment^49^—has been related to a vesicle pool size pathology.^50^ Quantifying the vesicle pool sizes of neurons treated with sorafenib and erastin therefore is a valuable addition to the common approaches on drug safety.

Key to the SOFA tool is a widespread approach for measuring synaptic vesicle pool sizes by use of pHluorin constructs.^51^ In brief, pHluorins are vesicular proteins, labeled by a pH-dependent fluorescence component. While fluorescence is quenched by the low pH inside the synaptic vesicle, upon a vesicle-membrane fusion event the molecules are exposed to the neutral pH of the synaptic cleft, emit fluorescence and can be recorded. With different external stimulation manoeuvers, the neurons are triggered to release synaptic vesicles of specific vesicle pools (e.g. readily releasable pool) whose size can then be quantified as the number of vesicles exocytosed upon stimulation.

Next to shedding light onto the effects of xCT inhibition on synaptic vesicle populations, our data highlight the importance of taking possible neuronal damage into account when designing new treatments for brain cancer. Approaching the concept of unintended and cytotoxic effects with the SOFA tool, we gain a comprehensive picture and can set morphological observations into context with metabolic and functional parameters.

## Results

Our first interest was to assess sorafenib’s effects on neuronal cell morphology, synaptic vesicle pool sizes, amino-acid secretome profiles and metabolic rates.

**Figure 1:**
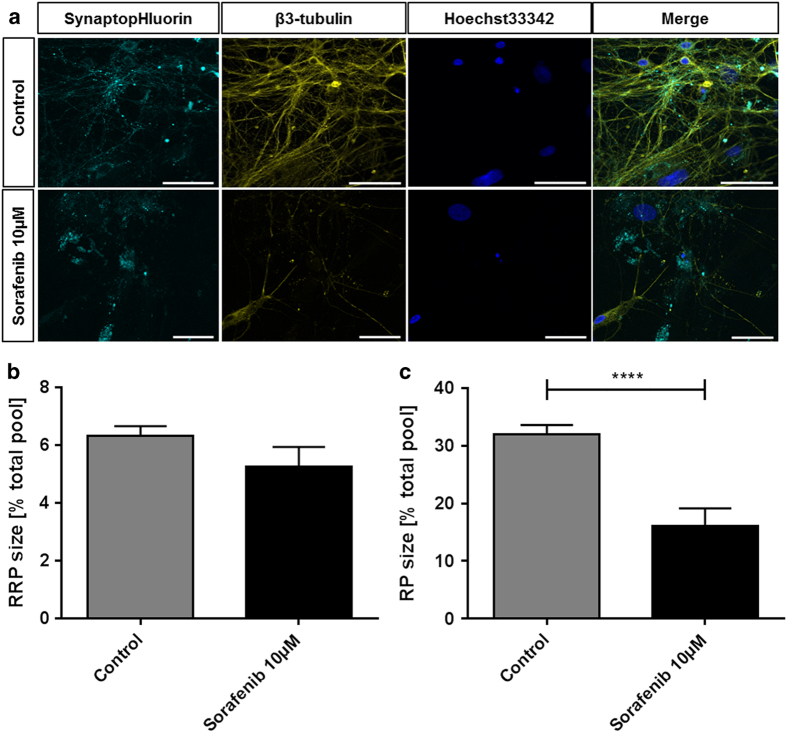
Sorafenib induces morphological alterations and reduces the synaptic vesicle pools in neurons. (**a**) Representative recordings of neurons treated with 10 *μ*M sorafenib (lower row) or controls (upper row) for 24 h. The samples were transfected with an EGFP-synaptobrevin2 (synaptopHIuorin) construct to visualize vesicles, stained against *β*3-tubulin to visualize the neurites and stained with Hoechst33342 that stains the nuclei. The scale bars span 50 *μ*m. (**b**) After a 24 h 10 *μ*M sorafenib treatment (or control), the readily releasable synaptic vesicle pool (RRP) size (released upon electrical stimulation with 40 stimuli at 20 Hz) was measured relative to the total vesicle population (perfusion with 50 mM ammonium chloride) for each synapse. The bar plot shows means with standard errors of the mean. Number of experiments: *n*=32 for control, *n*=7 for 10 *μ*M sorafenib; unpaired two-sided *t*-test, *P*=0.2013. (**c**) After a 24 h 10 *μ*M sorafenib treatment (or control), the synaptic vesicle recycling pool (RP) size (released upon electrical stimulation with 1200 stimuli at 40 Hz) was measured relative to the total vesicle population (perfusion with 50 mM ammonium chloride) for each synapse. The bar plot shows means with standard errors of the mean. Numbers of experiments: *n*=32 for control, *n*=7 for 10 *μ*M sorafenib; unpaired two-sided *t*-test, ****P=0.0002.

At the concentration of 10 *μ*M, that proved efficient in targeting tumor cells in Dixon *et al.*^8^, we found that neurons also suffered from sorafenib treatment. Figure 1a shows exemplary recordings of cultures treated with 10 *μ*M for 24 h. Compared to the co-localized vesicles and neurites seen in the control group, aggregates of synaptic vesicles are forming instead. The usual synaptic puncta staining pattern of the spH-transfected cells is lost. Also the extent to which the cytoskeleton is affected by the treatment with 10 *μ*M sorafenib is clearly visible. These features witness to a damage of the structure and morphology of the neurons in response to 10 *μ*M sorafenib treatment. With regard to synaptic function, the effect becomes even more visible: while basic synaptic architecture can still be maintained (readily releasable pool: control: 6.325±0.348%, *n*=32, sorafenib: 5.273±0.348%, *n*=7), the recycling pool is reduced from 31.98±1.642% (*n*=32) to only 16.04±3.11% (*n*=7) in the treated neurons (Figure 1b and c). In addition to this strong interference with synaptic vesicle recycling, electrical excitability, that is, the ability to induce synaptic vesicular release, was unusually weak in the treated group (data not shown), which also explains this experimental group’s lower sample size.

**Figure 2:**
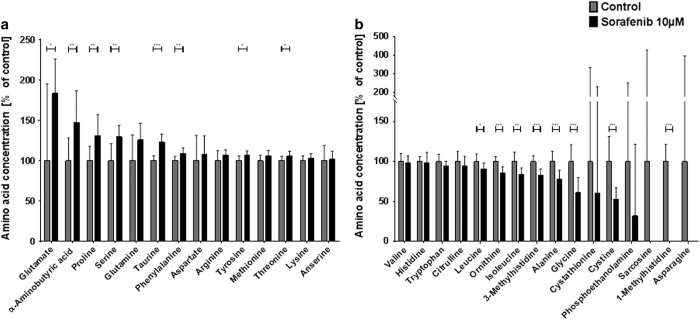
Sorafenib disturbs the extracellular amino acid profile of neurons. Primary hippocampus cultures were incubated with 10 *μ*M sorafenib (or control) for 24 h and their supernatant was collected and analyzed by high-performance liquid chromatography (HPLC). (**a**) Amino acids upregulated in response to 10 *μ*M sorafenib treatment with their concentration relative to the respective controls. Asterisks indicate the level of significance in an unpaired two-sided *t*-test. The bar plot shows means with standard deviations. Numbers of experiments: *n*=12 for control, *n*=8 for 10 *μ*M sorafenib. (**b**) Amino acids downregulated in response to 10 *μ*M sorafenib treatment with their concentration relative to the respective controls. Asterisks indicate the level of significance in an unpaired two-sided *t*-test. The bar plot shows means with standard deviations. Numbers of experiments: *n*=12 for control, *n*=8 for 10 *μ*M sorafenib. Levels of significance: *P<0.05, **P<0.01, ***P<0.001. A comprehensive table of all descriptive statistics and the analysis parameters can be found in Table 1.

In agreement with the morphologically and functionally apparent damage, the amino-acid profile of cultures treated with 10 *μ*M sorafenib was massively disturbed (Figure 2). We observed an almost doubled concentration of glutamate (183.49% of control±14.05%) and elevated concentrations of the amino acids proline, serine, glutamine, taurine, phenylalanine, aspartate, arginine, tyrosine, methionine and threonine (Figure 2a). A comprehensive list of amino-acid concentrations of all experimental groups and the corresponding statistical analysis are shown in Table 1. Amino-acid concentrations comparable to controls were found for lysine, asparagine, valine, histidine, tryptophan and citrulline, whereas concentrations of leucine, ornithine, isoleucine, 3-methylhistidine, alanine, glycine, cythathionine, cystine, sarcosine and 1-methylhistidine were found to be lower than in controls (Figure 2b).

Altogether, treatment with 10 *μ*M sorafenib for 24 h lead to enormous morphological and functional alterations, as well as to a disturbed amino-acid profile of the cultures. The damages were so strong that they became apparent in each of our viability assays.

**Figure 3:**
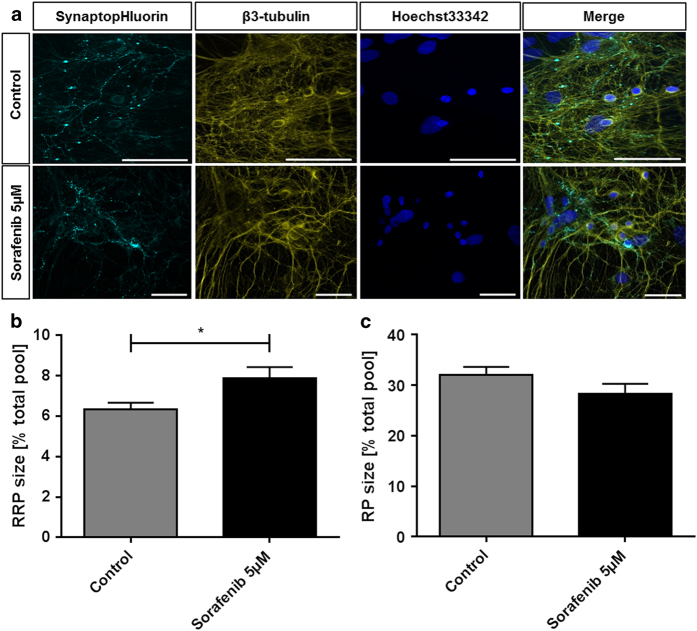
Low-dose sorafenib treatment does not affect neuronal morphology but impairs neuronal function by increasing the synaptic readily releasable pool. (**a**) Exemplary recordings of cells treated with 5 *μ*M sorafenib (lower row) or controls (upper row) for 24 h. The samples were transfected with EGFP-synaptobrevin2 (synaptopHIuorin) to visualize vesicles, stained against *β*3-tubulin to visualize the neurites and stained with Hoechst33342 that stains the nuclei. The scale bars span 50 *μ*m. (**b**) After a 24 h 5 *μ*M sorafenib treatment (or control), the readily releasable synaptic vesicle pool (RRP) size (released upon electrical stimulation with 40 stimuli at 20 Hz) was measured relative to the total vesicle population (perfusion with 50 mM ammonium chloride) for each synapse. The bar plot shows means with standard errors of the mean. Number of experiments: *n*=32 for control, *n*=21 for 5 *μ*M sorafenib; unpaired two-sided *t*-test, *P=0.018. (**c**) After a 24 h 5 *μ*M sorafenib treatment (or control), the synaptic vesicle recycling pool (RP) size (released upon electrical stimulation with 1200 stimuli at 40 Hz) was measured relative to the total vesicle population (perfusion with 50 mM ammonium chloride) for each synapse. The bar plot shows means with standard errors of the mean. Number of experiments: *n*=32 for control, *n*=21 for 5 *μ*M sorafenib; unpaired two-sided *t*-test, *P*=0.161.

In contrast to these devastating effects of a 10 *μ*M sorafenib treatment, our experiments show that treatment with only 5 *μ*M sorafenib—a concentration that is still efficient in reducing tumor cell growth^52^—did not affect the cell morphology (Figure 3a). Synapses and neurites align, and neither the pattern of vesicle staining nor the cytoskeleton or the cell nuclei is changed in their appearance compared to controls.

**Figure 4:**
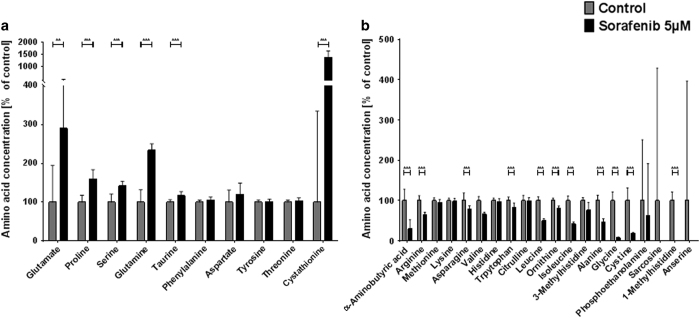
Low-dose sorafenib treatment disturbs the extracellular amino-acid profile of hippocampal cultures. Primary hippocampus cultures were incubated with 5 *μ*M sorafenib (or control) for 24 h and their supernatant was collected and analyzed by high-performance liquid chromatography (HPLC). (**a**) Amino acids upregulated in response to 5 *μ*M sorafenib treatment with their concentration relative to the respective controls. Asterisks indicate the level of significance in an unpaired two-sided *t*-test. The bar plot shows means with standard deviations. Number of experiments: *n*=12 for control, *n*=10 for 5 *μ*M sorafenib. (**b**) Amino acids downregulated in response to 5 *μ*M sorafenib treatment with their concentration relative to the respective controls. Asterisks indicate the level of significance in an unpaired two-sided *t*-test. The bar plot shows means with standard deviations. Number of experiments: *n*=12 for control, *n*=10 for 5 *μ*M sorafenib. Levels of significance: **P<0.01, ***P<0.001. A comprehensive table of all descriptive statistics and the analysis parameters can be found in Table 1.

Despite this seemingly intact morphology of the 5 *μ*M sorafenib-treated cultures, the extended analysis of functional and metabolic parameters still showed severe alterations. The general synaptic vesicle recycling cycle is not disturbed and with that the vesicular recycling pool is unaffected (sorafenib: 28.32±1.973%, *n*=21; control: 31.98±1.642%, *n*=32) (Figure 3b). The readily releasable pool (Figure 3c) on the other hand significantly grows from 6.325±0.3475% (*n*=32) to 7.860±0.057% (*n*=21) in the treated cells—an effect also found in neurons treated with tetrodotoxin.^53^ Such an increase in readily releasable pool size is accompanied by an increased number of docked vesicles and such a facilitated neurotransmitter release^53^ constitutes a serious intrusion of neuronal function. As it can be expected from the disturbed synaptic homeostasis, the amino-acid profile of cultures treated with 5 *μ*M sorafenib is also diversely affected by the treatment (Figure 4a and b). Similar to the cultures treated with 10 *μ*M, we find an almost three-fold rise of extracellular glutamate levels in the treated cultures compared to controls (290.48% of control±55.03%). The further amino-acid profile also shows the same up- and downregulation as the 10 *μ*M sorafenib-treated cultures. The only exception is the cystathionine, which is massively increased only in the 5 *μ*M sorafenib-treated cultures (10 *μ*M: 60.32±56.42%; 5 *μ*M: 1382.22±86.94%) and arginine, which is slightly upregulated after a 10 *μ*M sorafenib treatment, but downregulated after a 5 *μ*M sorafenib treatment (10 *μ*M: 106.80±1.95%; 5 *μ*M: 65.34±1.76%).

Although the lower concentration of 5 *μ*M sorafenib—in contrast to the 10 *μ*M concentration—did not suggest any damage in the morphological examination, the investigation of functional parameters and the amino-acid profile reveals an interference of the treatment with the sensitively regulated homeostasis of neuronal cultures and their function. This underlines the importance of an extension of neurotoxicity studies to also include these parameters.

With the previous results, it becomes clear that a treatment of glioblastoma cells *in situ*—surrounded by neurons—could affect proper neuronal function. As sorafenib binds to many different targets on the different cell types, we tried to isolate the effect mediated through the xCT-blockage pathway by comparing these outcomes to those obtained from the experiments with erastin, a different xCT inhibitor.

**Figure 5:**
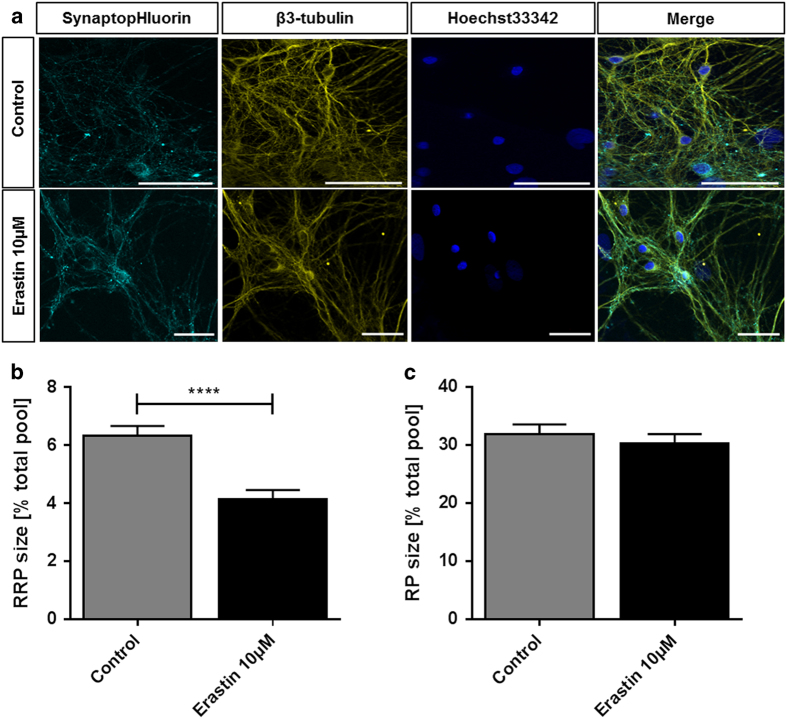
Erastin reduces readily releasable pool size but does not affect recycling pool size or the neuroskeletal architecture. (**a**) Representative recordings of cells treated with 10 *μ*M erastin (lower row) or controls (upper row) for 24 h. The samples were transfected with EGFP-synaptobrevin2 (synaptopHIuorin) to visualize vesicles, stained against *β*3-tubulin to visualize the neurites and stained with Hoechst33342 that stains the nuclei. The scale bars span 50 *μ*m. (**b**) After a 24 h 10 *μ*M erastin treatment (or control), the readily releasable synaptic vesicle pool (RRP) size (released upon electrical stimulation with 40 stimuli at 20 Hz) was measured relative to the total vesicle population (perfusion with 50 mM ammonium chloride) for each synapse. The bar plot shows means with standard errors of the mean. Number of experiments: *n*=32 for control, *n*=19 for 10 *μ*M erastin; unpaired two-sided *t*-test, ****P<0.0001. (**c**) After a 24 h 10 *μ*M erastin treatment (or control), the synaptic vesicle recycling pool (RP) size (released upon electrical stimulation with 1200 stimuli at 40 Hz) was measured relative to the total vesicle population (perfusion with 50 mM ammonium chloride) for each synapse. The bar plot shows means with standard errors of the mean. Number of experiments: *n*=32 for control, *n*=19 for 10 *μ*M erastin; unpaired two-sided *t*-test, *P*=0.512.

Despite the known efficacy of erastin on tumor cell lines,^8^ there was no damage visible in the morphological examination of the treated neuronal cultures. The overlay of neurites and vesicles that we observed in untreated controls is not changed after 24 h of 10 *μ*M erastin treatment (Figure 5a). Neither are the cytoskeletons or the cells’ nuclei deformed or damaged.

**Figure 6:**
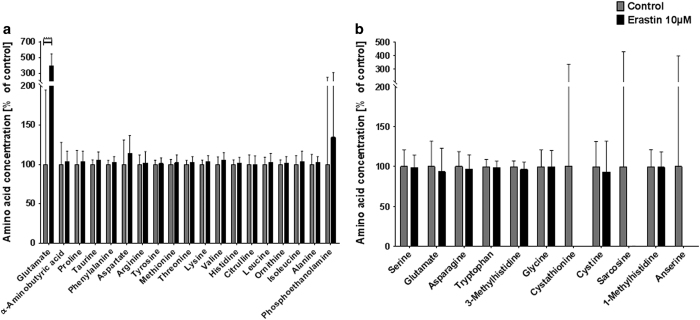
High-dose erastin treatment leads to critical extracellular glutamate release. Primary hippocampus cultures were incubated with 10 *μ*M erastin (or control) for 24 h and their supernatant was collected and analyzed by high-performance liquid chromatography (HPLC). (**a**) Amino acids upregulated in response to 10 *μ*M erastin treatment with their concentration relative to the respective controls. Asterisks indicate the level of significance in an unpaired two-sided *t*-test. The bar plot shows means with standard deviations. Number of experiments: *n*=12 for control, *n*=12 for 10 *μ*M erastin. (**b**) Amino acids downregulated in response to 10 *μ*M erastin treatment with their concentration relative to the respective controls. Asterisks indicate the level of significance in an unpaired two-sided *t*-test. The bar plot shows means with standard deviations. Number of experiments: *n*=12 for control, *n*=12 for 10 *μ*M erastin. Levels of significance: ***P<0.001. A comprehensive table of all descriptive statistics and the analysis parameters can be found in Table 1.

Erastin, next to xCT inhibition, also induces VDAC-1 opening, which upregulates cytosolic calcium concentration.^29^ The delicate calcium-dependent regulation of synaptic vesicle recycling^44^ responds to the erastin treatment with a readily releasable pool reduced by over one-third compared to controls (control: 6.325% of total pool±0.348%, *n*=32; 10 *μ*M erastin: 4.138±0.318%, *n*=19) (Figure 5b). The overall size of the vesicular recycling pool is not altered in the erastin-treated cultures (control: 31.98±1.642%, *n*=32; 10 *μ*M erastin: 30.35±1.609%, *n*=19) (Figure 5c). Similar to the selective modulation of presynaptic release probability (readily releasable pool^54,55^), the analysis of extracellular fluid brought out the same strong increase in glutamate levels (390.86% of control±43.45%) as the xCT inhibition with sorafenib, but brought out otherwise unchanged amino-acid concentrations (Figure 6a and b).

**Figure 7:**
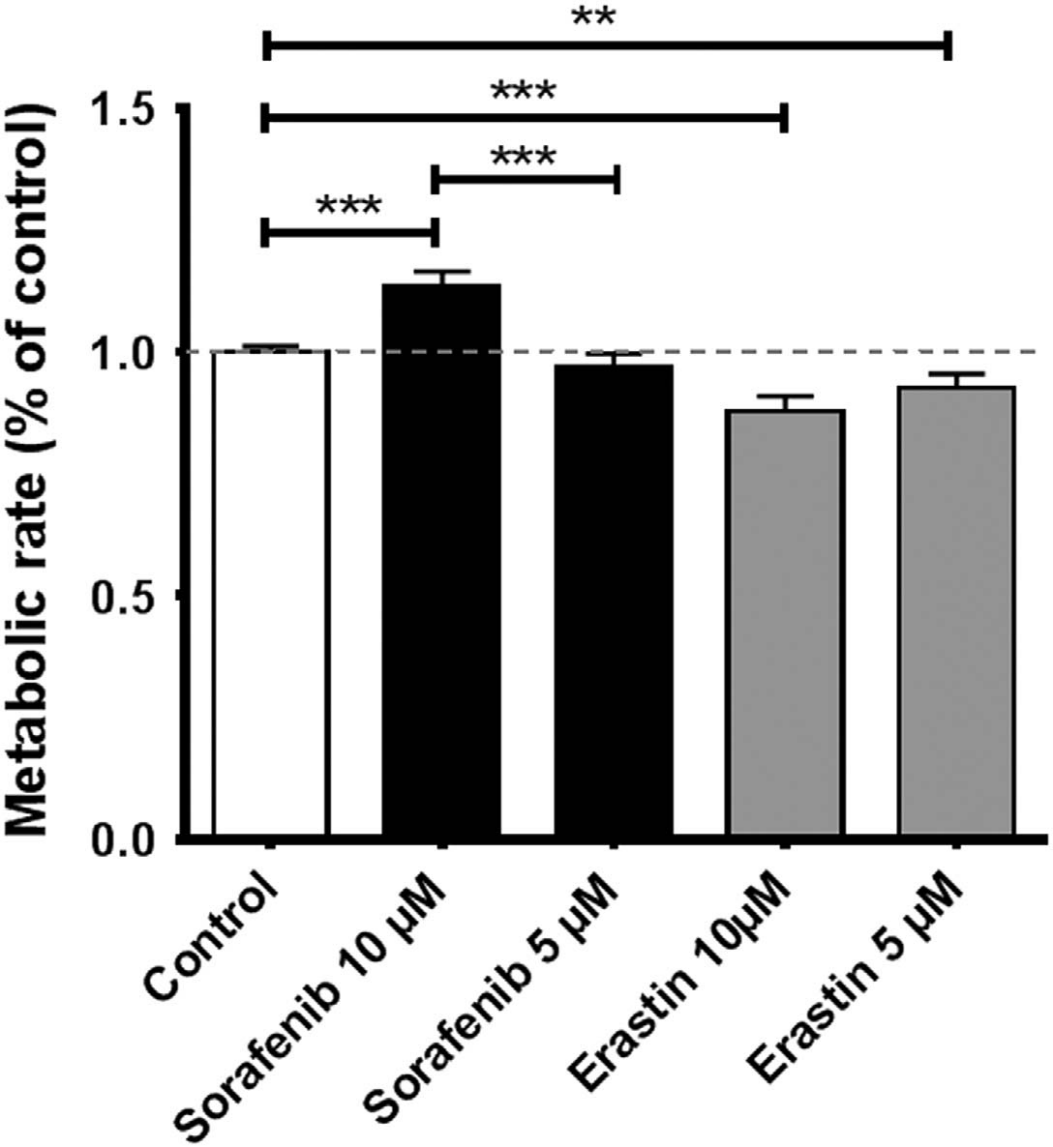
Metabolic rates are dose-dependently altered after sorafenib and erastin treatment. The MTT assay was performed using 3(4,5-dimethylthiazol)-2,5-diphenyltetrazolium (MTT) and detecting the rate of its metabolism to formazan in primary hippocampus cell culture. Formazan was detected measuring light absorption at 550 nm (wavelength correction 690 nm). The figure presents the pooled data of two experiments with more than six replications each, and individual controls. The data are normalized to respective controls and represent the mean with standard error of the mean. Statistical significance in two-sided *t*-test: **P<0.01, ***P<0.001.

After revealing the effects of sorafenib and erastin treatment on the extracellular amino-acid regulation, we turned to intracellular metabolism and investigated metabolic rates under the influence of both substances (and controls) using a MTT assay (Figure 7).

We found that despite the deteriorated morphology, cultures treated with 10 *μ*M sorafenib metabolized MTT with the highest rate of all tested groups (113.6% of control±12.5%, *P*<0.0001). The lower concentration of 5 *μ*M sorafenib had no effect on the cells’ metabolic rate compared to controls (96.7±2.9%, *P*=0.2320). In contrast to the even enhancing effect of sorafenib, erastin dose-dependently lowered the metabolic rate of the treated cells. Treatment with 5 *μ*M erastin slowed MTT metabolism to only 92.6±2.6% of the controls (*P*=0.0067) and treatment with 10 *μ*M erastin yielded a lowered rate of 87.7±2.7% of controls (*P*<0.0001).

## Discussion

Although sorafenib and erastin are xCT inhibitors, we surprisingly found that treatment with both substances increased the extracellular concentration of glutamate. In view of the importance of glutamate as a neurotransmitter and the complexity of its regulatory network,^56^ it seems clear that inhibition of the antiporter xCT system does not inevitably lead to a reduced extracellular glutamate concentration, at least when there are several different cell types in one culture, for example, astrocytes, neurons and microglia. Although further research is needed to determine what is the main origin of these increased glutamate levels, we hypothesize that inhibiting one part of the system regulating glutamate leads to cell stress and subsequently increases demand for cystine for glutathione generation. These imbalances in the glutathione pool are counteracted by other parts of this system, ultimately increasing the extracellular glutamate concentration.

Our data further show that neither sorafenib nor erastin leaves the mixed hippocampal cells unaffected. The 10 *μ*M sorafenib treatment massively disturbed neuronal morphology and function, and although MTT metabolism appeared healthy, even enhanced, the amino-acid homeostasis collapsed as it does, for example, also in ischemia.^57^

The lower concentrated 5 *μ*M sorafenib treatment did not affect neurons as severely. Although still showing an imbalanced amino-acid profile, both the synaptic vesicle recycling pool and cell morphology were preserved. The presynaptic availability of readily releasable vesicles, however, was modified at the lower concentration of 5 *μ*M sorafenib. The interference with the sensitive regulation of synaptic vesicles is thought to be the underlying cause of many psychiatric disorders^45^ and has been studied in the context of a wide range of neurotoxins.^53,54^

The reference experiments with erastin showed that even though MTT metabolism is decreased in the erastin-treated cultures, the effects of the substance on cell morphology, synaptic vesicle recycling and the interference with vesicle docking (RRP) are comparable to sorafenib. Yet, erastin did not affect the cultures’ extracellular amino-acid profile, which suggests that this part of sorafenib’s effects might be due to off-target effects.

These results prove that the sole examination of morphological parameters—as it is common in when evaluating new chemotherapeutic agents^37,38,39,58^—might be misleading in the context of neurons.

Morphological parameters are severely affected by a 10 *μ*M sorafenib treatment, but also with the seemingly healthy morphology at 5 *μ*M, the extracellular amino-acid profile and presynaptic function are still disturbed. Erastin, too, interferes with neuronal function while not showing any signs of damage when neurons are investigated morphologically.

As the MTT assay is very sensitive to cellular metabolism,^59^ its results alone are not suited to preclude toxicity, either. The enhancement of glycolysis in sorafenib-treated cultures^60^ might be responsible for the high MTT metabolism and the loss of synaptic vesicles^45^ even though morphological examination clearly shows signs of toxicity for the 10 *μ*M concentration. The relatively low MTT metabolism rate in erastin-treated cells on the other hand might not be a sign of a weak viability but rather is the consequence of erastin’s binding to mitochondrial membrane proteins (mPTP and VDAC-1^29^).

With that, the strong changes in cell metabolism and the extracellular amino-acid regulation of sorafenib might be mediated by xCT-independent targets. In contrast, the interference with presynaptic vesicle docking, indeed, seems to be a result of xCT inhibition, as sorafenib and erastin both interfere with the delicate regulatory system^45^ mediating the recruitment of synaptic vesicles to the readily releasable pool.

Our findings of this first study on the effects of xCT inhibitors on healthy neuronal cells show that proper neuronal function cannot be deduced solely from morphological or metabolic parameters. We applied the SOFA tool that allows an in-depth analysis of neuronal function at the synaptic level. This study also provides evidence for the hypothesis that—in addition to their desirable effect on tumor cells—sorafenib and erastin interfere with neuronal function and extracellular homeostasis, which should be taken into account when developing future chemotherapeutics on the basis of xCT inhibitors.

## Materials and methods

If not stated otherwise, all chemicals were purchased from Sigma-Aldrich (Taufkirchen, Germany). The experiments were conducted in accordance with the local ethic guidelines of the state of Bavaria, Germany. Primary hippocampal cultures were prepared from newborn Wistar rats (Charles River, Wilmington, MA, USA) as described before.^61,62^ In short, hippocampi were removed, washed and dissociated with trypsin. After a centrifugation step they were plated on precoated glass cover slips and incubated with medium.

On the third day in culture, cells were transfected with synapto-pHIuorin (spH) (PlasmidFactory, Bielefeld, Germany)^63^ by a modified calcium phosphate method.^64^ For that, the cells were incubated with transfection solution (containing 60 *μ*l DNA, 60 *μ*l CaCl_2_, 480 *μ*l H_2_O, 2×BBS 600 *μ*l, 10.8 ml NBA) for 30 min, allowing calcium chloride molecules to form precipitates. Afterwards, cells were washed (HBSS) and incubated (37 °C, 5% CO_2_, 95% rH) for 30 days in medium.

Prior to the experiments, the cells’ medium was supplemented with 10 *μ*M sorafenib, 5 *μ*M sorafenib or 10 *μ*M erastin for 24 h. These concentrations were found to efficiently target glioblastoma cell lines in previous studies.^8^ For control, cells were incubated with the respective volume of the vehicle dimethyl sulfoxide (DMSO) (Carl Roth, Karlsruhe, Germany).

### Immunofluorescence staining

For an assessment of the cells’ morphology, the cultures were fixated and then antibody-stained and imaged.

In detail, the cultures were washed in PBS and fixated in 4% paraformaldehyde for 15 min. The fixated cells were washed twice with PBS and then permeabilized (10 min in 1×PBS with 0.3% Triton-X). The samples were incubated in blocking solution containing 1×PBS, 0.3% Triton-X and 3% fetal calf serum (Invitrogen, Taufkirchen, Germany). Primary *β*-tubulin III antibody (G7121, mouse monoclonal, Promega, Madison, WI, USA) was diluted in blocking solution and left on the cells for 24 h at 4 °C. The samples were then washed thrice in blocking solution and subsequently incubated for 24 h at 4 °C with the secondary antibody (Alexa Fluor 568 goat anti-mouse IgG, A-11004 Invitrogen, Taufkirchen, Germany), diluted 1:1000 in blocking solution. After washing thrice with blocking solution, the nuclei were stained with Hoechst33342, diluted 1:5000 in PBS. Images were taken with an ApoTome and the Zen Software (Zeiss, Oberkochen, Germany).

### Synaptic optogenetic function analysis tool

After the sorafenib, erastin or control incubation for 24 h, the coverslips for the synaptic vesicle pool size measurement were placed in perfusion chambers, covered with 500 *μ*l of imaging buffer (in mM: 144 NaCl, 2.5 KCl, 10 glucose, 10 HEPES, 2.5 CaCl_2_ and 2.5 MgCl_2_, pH=7.5, supplemented with 80 nM concanamycin A and the corresponding treatment substance).

The fluorescence signal of the spH-transfected neuronal cell culture was recorded with a Nikon (Minato, Tokyo, Japan) TI-Eclipse inverted fluorescence microscope, equipped with a ×60, 1.2 NA water immersion objective. To stay in focus during perfusion, a Nikon Perfect Focus System was used. The fluorescent probes were excited by a Nikon Intensilight C-HGFI in a range of wavelengths 455–485 nm. The emitted light was recorded by a −90 °C water-cooled EM-CCD camera (iXonEM DU-885, Andor, Belfast, Northern Ireland), after passing an emission band-pass filter (Semrock, Rochester, NY, USA), ranging from 500 to 545 nm. The dichroic mirror had a cutoff wave length of 495 nm. A constant perfusion rate with imaging buffer (0.5 ml/min) during the recordings was ensured using a piezo-controlled perfusion system (SF-77B, Warner Instruments, Hamden, CT, USA). Constant fluid levels were maintained by using a fluid level control and a peristaltic pump (MPCU-3, Lorenz Messgerätebau, Katlenburg-Lindau, Germany).

The neurons were stimulated to release the different synaptic vesicle pools by electric field stimulation (51 mA for 1 ms, alternating polarity) delivered through two parallel platinum electrodes, spanning a distance of 10 mm. Stimulation (STG 4008, Multichannel Systems, Reutlingen, Germany) was performed in combination with a stimulus isolator (World Precision Instruments, Sarasota, FL, USA).

Recordings were exported into tagged image file (tif) format, containing 512×512 pixels of 16-bit monochromatic pixel values. Release of synaptic vesicles was electrically stimulated with 40 pulses at 20 Hz, evoking exocytosis of the readily releasable pool and 1200 pulses at 40 Hz, evoking exocytosis of the recycling pool.^65^ The total pool was visualized by perfusion with alkaline imaging buffer containing additional ammonium chloride (50 mM). Images were acquired with an exposure time of 150 ms at a frame rate of 5 Hz.

The recorded image stacks were imported into MatLab (The Mathworks Inc., Natick, MA, USA) and further analyzed with custom-written routines. After visual inspection, measurements with no response to electrical stimuli were excluded, since a lack of excitability was seen as a lack of vitality. Synapses were then automatically detected by background-determination-based feature point detection^66^ and fluorescence traces for each cell were cleared from baseline and normalized to the intensity of the total vesicle pool. The relative size of the different released vesicle pools was calculated from the stepwise increase of synaptic fluorescence upon stimulation and its linear proportionality to the relative number of released vesicles.^50^

### Amino-acid profiling

Metabolic assays were performed with cultures containing 10 mM glucose and 2–4 mM glutamine. To measure consumption and secretion of amino acids, cell supernatants were collected after 24 h with erastin or sorafenib and were measured by using high-performance liquid chromatography (HPLC). Amino acids were analyzed by ion-exchange chromatography and post-column ninhydrin derivatization technique using a fully automated amino-acid analyzer (Biochrom 30+, Laborservice Onken, Gründau, Germany). For the amino-acid analysis, 100 *μ*l of sample was deproteinized with 100 *μ*l of 10% sulphosalicylicacids. Afterwards, 20 *μ*l of this supernatant was then loaded by the autosampler into a cation-exchange resin-filled column.

### Cell metabolism analysis

To assess the treated cells’ metabolic rate as a sign of cell viability, we performed a 3(4,5 dimethylthiazol)-2,5 diphenyltetrazolium (MTT) assay as described by Sehm *et al.*^31^ After 24 h, incubation with either 5 or 10 *μ*M sorafenib or erastin, cells were incubated with freshly made MTT solution (Roth, Karlsruhe, Germany) (5 mg/ml) for 4 h at 37 °C, 5% CO_2_. We used 100 *μ*l isopropanol, supplemented with 0.1 N HCl for the following cell lysis. The optical density of each well was determined using the microplate reader Tecan Infinite F50 (Crailsheim, Germany) set to 550 nm (wavelength correction set to 690 nm).

### Statistical analysis

If not stated otherwise, the data are given as mean with standard error of the mean and were analyzed with an unpaired two-sided *t*-test with an alpha of 0.05.

## Figures and Tables

**Table 1:** Amino-acid profiles of mixed neuronal cultures after treatment with xCT-inhibitors sorafenib and erastin

*Amino acid*	*Control*	*Sorafenib 10 μM*	*Sorafenib 5 μM*	*Erastin 10 μM*
	n*=12*	n*=8*	n*=10*	n*=12*
Glutamate	100.00**±**27.40^~^	**183.49±14.05,** ***P*****=0.03218**^**^**^	**290.48±58.01,** ***P*****=0.00518**^**^**^	**390.86±43.45**, ***P*****=0.00002**^**^**^
α-Aminobutyric acid	100.00**±**8.19^~^	**147.11±13.03,** ***P*****=0.00593**^**^**^	**30.04±7.08,** ***P*****<0.00001**	103.69**±**3.75, *P*=0.68846^~^
Proline	100.00**±**5.19^~^	**130.82±8.73,** ***P*****=0.00590**^**^**^	**159.98±7.96,** ***P*****<0.00001**^**^**^	103.59**±**3.78, *P*=0.58784^~^
Serine	100.00**±**6.09^~^	**129.33±4.79,** ***P*****=0.00306**^**^**^	**142.10±4.03,** ***P*****=0.00002**^**^**^	98.35**±**4.46, *P*=0.83189^~^
Glutamine	100.00**±**9.20^~^	125.72**±**6.85, *P*=0.05996^**^**^	**234.19±5.44,** ***P*****<0.00001**^**^**^	93.63**±**8.15, *P*=0.61632^~^
Taurine	100.00**±**1.78^~^	**122.65±3.39,** ***P*****=0.00001**^**^**^	**117.07±3.45,** ***P*****=0.00016**^**~**^	105.78**±**2.85, *P*=0.10965^~^
Phenylalanine	100.00**±**1.59^~^	**109.13±2.25,** ***P*****=0.00392**^**~**^	105.10**±**2.72, *P*=0.10772^~^	102.58**±**2.07, *P*=0.34757^~^
Aspartate	100.00**±**9.08^~^	108.21**±**7.46, *P*=0.53348^~^	119.68**±**9.76, *P*=0.15611^~^	114.01**±**6.37, *P*=0.22609^~^
Arginine	100.00**±**3.54^~^	106.80**±**2.15, *P*=0.16980^~^	**65.34±1.76,** ***P*****<0.00001**	101.91**±**4.08, *P*=0.73408^~^
Tyrosine	100.00**±**1.65^~^	**106.33±1.95,** ***P*****=0.02756**^**~**^	101.25**±**2.34, *P*=0.65815^~^	101.57**±**2.02, *P*=0.56312^~^
Methionine	100.00**±**1.91^~^	106.28**±**2.18, p=0.05184^~^	95.15**±**2.32, *P*=0.11844^~^	102.35**±**2.83, *P*=0.51026^~^
Threonine	100.00**±**1.57^~^	**105.80±1.95,** ***P*****=0.03645**^**~**^	103.34**±**2.57, *P*=0.26301^~^	102.72**±**2.04, *P*=0.31588^~^
Lysine	100.00**±**1.74^~^	102.66**±**1.98, *P*=0.34607^~^	98.53**±**2.34, *P*=0.61395^~^	103.83**±**2.17, *P*=0.19347^~^
Asparagine	100.00**±**5.47^~^	101.53**±**3.49, *P*=0.83874^~^	**79.08±2.62,** ***P*****=0.00416**	96.87**±**4.95, *P*=0.68151^~^
Valine	100.00**±**2.90^~^	98.67**±**2.88, *P*=0.76430^~^	**66.59±1.42,** ***P*****<0.00001**	105.53**±**2.75, *P*=0.18892^~^
Histidine	100.00**±**1.77^~^	98.23**±**4.34, *P*=0.68729^~^	97.55**±**2.24, *P*=0.39382^~^	101.44**±**2.15, *P*=0.61966^~^
Tryptophan	100.00**±**2.57^~^	94.24**±**2.03, *P*=0.12967^~^	**83.57±3.30,** ***p*****=0.00073**^**~**^	98.34**±**2.34, *P*=0.64500^~^
Citrulline	100.00**±**3.63^~^	94.24**±**4.05, *P*=0.32358^~^	99.64**±**2.45, *P*=0.93849^~^	100.26**±**2.99, *P*=0.95762^~^
Leucine	100.00**±**2.76^~^	**90.58±2.53,** ***P*****=0.03185**^**~**^	**50.33±1.43,** ***P*****<0.00001**	102.62**±**3.19, *P*=0.55057^~^
Ornithine	100.00**±**1.76^~^	**85.73±2.48,** ***P*****=0.00018**^**~**^	**80.98±1.72,** ***P*****<0.00001**^**~**^	101.70**±**2.32, *P*=0.57471^~^
Isoleucine	100.00**±**3.39^~^	**83.65±2.82,** ***P*****=0.00335**^**~**^	**42.37±1.35,** ***P*****<0.00001**	103.74**±**3.72, *P*=0.47555^~^
3-Methylhistidine	100.00**±**2.10^~^	**82.76±2.71,** ***P*****=0.00010**^**~**^	**76.14±6.14,** ***P*****=0.00078**	96.58**±**2.55, *P*=0.32288^~^
Alanine	100.00**±**3.78^~^	**77.66±3.79,** ***P*****=0.00100**	**47.56±2.32,** ***P*****<0.00001**	102.62**±**2.07, *P*=0.55292^~^
Glycine	100.00**±**6.09^~^	**61.26±6.24,** ***P*****=0.00055**	**7.76±0.61,** ***P*****<0.00001**	99.95**±**5.61, *P*=0.99552^~^
Cystathionine	100.00**±**67.67^~^	60.32**±**56.42, *P*=0.68645	**1382.22±86.94,** ***P*****<0.00001**^**^**^	0.00**±**0.00, *P*=0.15367
Cystine	100.00**±**9.07^~^	**52.73±4.79,** ***P*****=0.00092**	**18.61±0.79,** ***P*****<0.00001**	93.20**±**10.72, *P*=0.64189^~^
Phosphoethanolamine	100.00**±**43.56^~^	31.76**±**29.71, *P*=0.26730	61.69**±**41.13, *P*=0.53549	134.64**±**49.00, *P*=0.61143^
Sarcosine	100.00**±**94.84^~^	0.00**±**0.00, *P*=0.40485	0.00**±**0.00, *P*=0.34924	0.08**±**0.08, *P*=0.30354
1-Methylhistidine	100.00**±**6.14^~^	**0.00±0.00,** ***P*****<0.00001**	**0.00±0.00,** ***P*****<0.00001**	99.10**±**5.38, *P*=0.91462^~^
Anserine	100.00**±**85.61^~^	0.00**±**0.00, *P*=0.35714	0.00**±**0.00, *P*=0.30092	0.00**±**0.00, *P*=0.25525

The levels of amino acids and metabolites in extracellular fluid are changed after treatment with different concentrations of sorafenib and erastin. Presented values are given as percent of untreated controls. The values are shown as mean ± standard error of the mean (SEM) and with *P*-values for two-sided *t*-tests. Upregulated amino acids are marked with ^; medium regulated amino acids (80–120% of controls) are marked with ^~^; downregulated amino acids are unmarked; alterations that are statistically significant are highlighted in bold font.
